# Decision Support System for Prioritizing Self-Assurance of Academic Writing Based on Applied Linguistics

**DOI:** 10.3389/fpsyg.2022.784508

**Published:** 2022-03-16

**Authors:** Yancheng Yang, Shah Nazir

**Affiliations:** ^1^Henan University of Science and Technology, Luoyang, China; ^2^Luoyang Institute of Science and Technology, Luoyang, China; ^3^Hunan University, Changsha, China; ^4^Department of Computer Science, University of Swabi, Swabi, Pakistan

**Keywords:** self-assurance, academic writing, applied linguistics, DSS, Language

## Abstract

Based on applied linguistics, this study looked at the decision support system (DSS) for emphasizing self-assurance in academic writing. From a generic perspective, academic writing has been considered both a process and a product. It has highlighted the planning composite processes, editing, composing, revising, and assessment, which depend upon the familiarity of someone with confidence in their capability for engagement in these activities. As a product, it has focused on the writing results through the product’s characteristics. These contain specific content areas in acceptable depth and well-structured technical vocabulary. Higher education aims to support students in optimizing their potential for achieving satisfactory outcomes. For example, the assessment of grades involves academic writing, contributing to the degree course classification. Students have differences in many respects, such as expectations, background knowledge, and study and learning approaches. There were varying students’ beliefs about what academic writing is for evaluation. Modern-day motivations and theories highlight the significance of students’ confidence in their studies. The role of high confidence can support students to apply more effort toward setting challenging goals. Students may find it more difficult to succeed in higher education if they lack confidence in their academic writing abilities. A DSS has many applications in diverse areas and can play a significant role in the ranking and prioritization process. The current study has considered the DSS for prioritizing self-assurance in academic writing based on applied linguistics. Various criteria were considered for the evaluation of the research. The Super Decision software was used for the experimental process of the proposed research. The results of the study show the effectiveness of the proposed research.

## Introduction

Higher education seeks to help students to reach their full potential and achieve good results. Academic writing, for example, is used to assess grades and contribute to the classification of degree courses. Expectations, baseline knowledge, and study and learning practices are all different across students. Students’ perceptions of what constitutes academic writing for evaluation were diverse. The importance of students’ confidence in their studies is highlighted in modern motives and ideas. High self-esteem can encourage pupils to put in more effort toward achieving difficult goals. If students are unsure about their academic writing talents, it may be more difficult for them to succeed in higher education. Language reliability of English as a second language (ESL) and English as a foreign language (EFL) has been considered one of the essential considerations in the development and evaluation of educational assessment. Studies in these areas have presented that many factors can affect the reliability assessment of these ESL and EFL (Barkaoui, 2007). Various studies have been considered since the last decade for the identification of score reliability and variability in assessing ESL and EFL. Theories of motivation were presented to show the students the important role of confidence. For gaining insights, Zotzmann and Sheldrake (2021) have surveyed 122 students of applied linguistics. The study presented that language one and language two students reported identical Bachelor of Arts (BA) grades, but language two students with lower average Master of the Arts (MA) grades. Particularly, lower confidence for their MA studies overall, lower confidence for English academic writing, fewer positive beliefs regarding effort about writing, and higher belief in writing intricate transmission. Among all the students, the MA grades reported confidence for the writing of academic in English positively correlated with belief concerning effort in writing, but negatively correlated with writing belief concerning transmission.

Marti et al. (2019) presented a study to investigate nativeness role and knowledge on reporting practices in writing, concerning the patterns to use and stance construction. Four research corpora articles in the field of applied linguistics of native English writers, Turkish non-native expert writers, Turkish novice non-native English writers, and novice English writers were compared. An analysis was done based on corpus for exploring the features of the sourced research reports, such as verb controlling clauses focus on subject type, reporting type, and reference type. The research’s findings elaborated that the expertise level is a significant part of disciplinary writing as native and non-native expert writers.

Different research works related to language behavior have been carried out based on cultures from a sociolinguistics perspective. Shin et al. (2017) have identified the language behavior effects of lecturers from various cultures in the system of e-learning. The study is based on the experimental results of the linguistics behaviors survey between Korean and Japanese from sociolinguistics perspectives. Cognitive ability has been considered one of the significant thinking modes in a language. Li (2017) analyzed the English for science and technology from three domains on the basis of cognitive linguistics. These domains include semantic level, lexical level, and grammatical level. Broad range applications of metaphor contribute more to the coherence and logicality of English for science and technology and facilitate as reference for science and technology students to enhance their proficiency of English. Language and culture are essential, and for students of Chinese colleges who are learning English, a learner of a foreign language has to learn the language in both English culture and Chinese culture. Yang et al. (2020), Yanping and Zhenhua (2020) described incorporating Chinese culture strategies into college English teaching in the college of authors. They demonstrated that it is desirable to integrate Chinese culture into English learning.

Ren (2021) has compared the level to which the lexical bundles from applied linguistics and pharmaceutical science from research articles are presented. The examination of lexical bundles across the discourse functions for exploring the probable relations between variability and function is done. The experimental results of the study revealed that variability describes disciplinary variation, which bundles from the articles of applied linguistics as complete less fixed. Additionally, the discourse functions are identified to be thoroughly related to variability. Tsepilova and Mikhaleva (2015) have considered learning to understand and use formulaic language as an indicator of their pragmatic skills. Empirical data were collected and examined to identify the current extent of the learners’ pragmatic skills and to disclose issues faced in the culture-specific communication situation. Activities of the classroom for the development of pragmatic skills have been considered for the suggestion. The contribution of the proposed study is given below:

• To devise a DSS in early decision-making based on the defined multicriteria for prioritizing self-assurance of academic writing based on applied linguistics.
• To propose fundamental factors (criteria) and available alternatives for the self-assurance of academic writing were considered from the literature.
• To simulate the process of the proposed research and to show the effectiveness of the proposed research in the form of experimental results.

## Literature Review

Various studies have been presented in the literature associated with language and its related culture. But no associated study which support the early decision-making for prioritizing self-assurance in academic writing based on applied linguistics has been found. Farsani et al. (2021) analyzed and coded procedural and methodological collaboration of 3,992 articles published on linguistics in the 18 prominent journals from 2009 to 2018. They classified the articles and showed that 4.5% of the articles were non-empirical while the rest were empirical. In the empirical studies, 42.6% were quantitative, 25.9% were mixed method research studies, and 24.9% were qualitative. The systematic literature showed a higher impact of citation compared to other research approaches. However, no significant difference exists between the citations number of the three techniques, including qualitative, quantitative, and mixed methods. General coloration, international collaboration, and multidisciplinary collaboration showed high significance in the articles that were quantitative. The applied linguistics researchers with a high rate of collaboration were shown in the disciplines of education and psychology. Chen and Hu (2020) reported a corpus-based study on linguistic expressions of a surprise based on 160 research articles of applied linguistics. The study presented a frame semantics perception on surprise as a knowledge emotion and implemented a fine-grained, frame-based analytical framework for examining diachronic trends in the surprise use markers and their occurrences with other meta-discourse. Compared with the existing studies, the binary logistic regressions showed that research published more recently were 2.16 times more possible for expressing surprises triggered by previous knowledge, 2.37 times more possible for expressing surprises without providing an explanation, and 2.28 times more possible for indicating surprises deprived of resolving them. Explanations were given to the results in terms of the heuristic nature of surprise and growing pressure on academics to promote their research strategically. Bartlett (2020) presented the idea of linguistic dynamism from the evolutionary and materialist perception. The study described the evolution of human traits that account for the variability of response within the explicit context and the level to which such variability is operative to safeguard the system integrity to allow emergence and consider adaptive behavior. The study has presented two processes: higher orders of extension and abstraction in the system of language and an upsurge in the meaning potential of the system.

Yamaguchi and Koyama (2009) presented a native grammarian’s view of Japanese, which was predicted in opposition to the analysis of the language according to the notions of “Western linguistics.” Riemer (2019) explored the “political” dimensions of the cognitive linguistics movement and deliberated with Ronald Langacker and George Lakoff. Cognitive linguistics is explored with the perspective of institutional politics required for disciplinary survival in a changing system of United States higher education, along with the wider socio-political situations of the field origins and development. Longhi (2021) presented a response to the forensic science digital transformation through the application of tool-based linguistic analysis which was incorporated into the prototype of digital humanities. With the support of digital methods, scientific modeling was done for digital text analysis. The role of computer science, along with linguistics skills, are apparent for supporting the analysis of criminal acts. Su et al. (2021) presented an approach of local grammar for investigating diachronically discourse acts of text in academic, aiming to offer a supportive approach to studies of diachronic academic discourse. The proposed approach of local grammar is revealed with a study exploring the changing patterns of exemplification in linguistics research articles, showing that the linguistic academic writers tend to represent in explicit and simpler ways. Schwarz and Hamman-Ortiz (2020) presented an overview of the empirical scholarship since the last decade, taking how teacher training in systemic functional linguistics theory and pedagogy influences elementary English learners’ disciplinary and writing learning outcomes. Four key themes which describes the possibility for systemic functional linguistics informed pedagogies for supporting elementary English learners in mastering the academic language and literacy skills were considered from this review, composing a genre-specific text, developing critical language awareness, and learning across academic disciplines.

Janssen and Schlaud (1999) conducted an exemplary study at SAP AG – a software solution, Germany. The author interviewed various English native speakers to learn to know what issues they face when writing in their native language out of the country for international readers. Liu (2009) has presented a study to identify the organizational culture effect on language classrooms at a new college. The study initially analyzed the organizational culture’s information, identifies its features, and then described the effects of the host educational background. Based on this, the study examined the effects of organizational culture on classroom culture concerning English language learning and teaching in China. Lin and Lee (2009) surveyed fuzzy linguistics through the centroid and signed distance method. The proposed approach is different from conventional survey algorithms through questionnaire rating by linguistic variables. Keeping the imprecise nature, the study has employed a fuzzy sense of sampling for expressing the level of the interviewee’s feelings according to his own idea. The signed distance method has been used to re-model the previous process for assessment analysis reliability and effectiveness.

The Internet has been considered a good-looking platform of cultural exchange in the literature and language intelligent teaching system, especially for foreigners to easily understand cultural background. The traditional teaching system is appropriate for native cultural backgrounds, but due to the limited time required, the conventional education facilities require extreme student caring capacity. Duan (2015) developed literature teaching and intelligent language modules in the environment of the network. Along with the related developments of a hardware system, the rising appropriateness of language learners has been described for the effects of learning. Cheng and Chiu (2018) presented a case study that focused on the improvements made during a lesson unit on descriptive writing. The study results showed that two pre-intermediate level writers described increased control in the usage of interpersonal, conceptual, and textual resources related to the descriptive genre.

## Decision Support System for Prioritizing Self-Assurance of Academic Writing Based on Applied Linguistics

People in pattern recognition applications have encountered various issues. Mother tongue transfer is one of the predictable applications in English translation. It has a significant effect on the learning and translation of foreign languages. Four forms of language transfer have mostly resided, including avoidance, overuse, convenience, and misuse. With the recognizable differences between English and Chinese culture, negative transfer of mother tongue cannot be escaped. Shi (2021) presented a study on the culture transfer of machine language translation through pattern recognition. Bahrami et al. (2015) addressed the cultural impartiality among the users and the Wikipedia editors in the representation of the Islamic world. The qualitative content analysis approach has been used in the research and deductive category application during a coded instruction containing fourteen factors. These factors include nine references and five dichotomous categories which have astonishingly described the great extent of impartiality culture among the English and Persian users of Wikipedia in the Muslim world for the editing process of text. Brock (2017) described two ways of raising the description of humor complexity. In the first one, central humor theories were considered in the complex analysis of humor phenomena in the linguistic disciplines. In the second way, the incorporation of aspects covered by diverse humor theories in complex models of communication. The research studied it as a central task of linguistics for providing other disciplines of humor research with the analytics that linguistic analysis can suggest. Sharifian (2017) presented a study for contributing to scholarly efforts to clarify the claims made by the early proponents of linguistic relativity. Cultural linguistics areas that are developed are presented and described the multidisciplinary fields of research.

**FIGURE 1:**
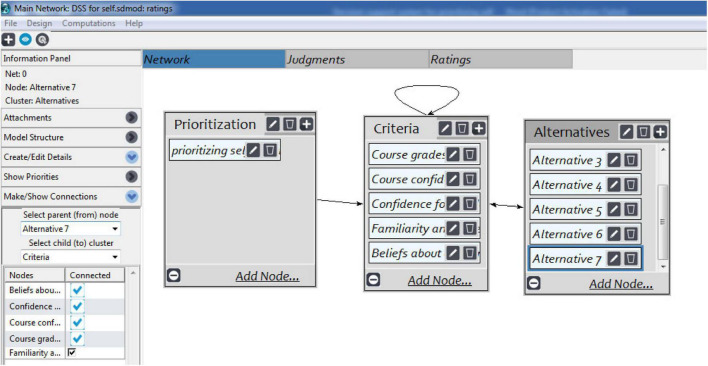
Process of the proposed research for prioritization, criteria, and alternatives. Permission to reproduce images of Super Decisions has been given by Creative Decisions Foundation.

Numerous applications of DSSs are available for diverse domains (Nazir et al., 2016; Yang et al., 2020; He et al., 2021; Sun et al., 2021). These applications are working according to criteria for assessing alternatives. The assessment is mostly done according to defined criteria, mostly multicriteria (Yang et al., 2020; Zhang et al., 2020; Sun et al., 2021). The approach used in the current research has the advantages it works in situation of complexity and vagueness. The Super Decisions tool has been used for the experimental process of the proposed research work. The limitation of the proposed work is that very few features have been considered. However, in future, an additional number of features will be considered. Figure 1 describes the process of three levels that is prioritization, criteria, and alternatives. The criteria defined for the proposed research were considered from the literature (Zotzmann and Sheldrake, 2021). The criteria included course grades, course confidence, confidence of academic writing, familiarities and ease with assessment genres, and belief about academic writing. Seven alternatives were available for the prioritization process and to select the top priority. These alternatives were named as “alternative 1,” “alternative 2,” and so on.

**FIGURE 2:**
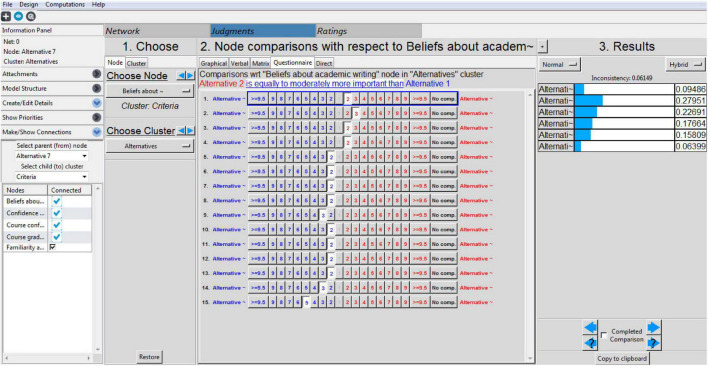
Comparison process of alternative based on the defined criteria. Permission to reproduce images of Super Decisions has been given by Creative Decisions Foundation.

Once the three levels were defined in the tool, the process of pairwise comparisons started. The comparisons were made for the criteria and alternatives. Figure 2 represents the comparison process for one option based on the defined criteria. The same process was followed for the rest of the alternatives and their criteria.

Once the process of comparisons has been completed, all the normalized values were brought into the unweighted supermatrix. Table 1 shows the unweighted supermatrix.

**TABLE 1:** Unweighted supermatrix.

	Alterna	Alterna	Alterna	Alterna	Alterna	Alterna	Alterna	Beliefs	Confide	Course	Course	Familia	priorit
Alterna	0.00000	0.00000	0.00000	0.00000	0.00000	0.00000	0.00000	0.09486	0.06993	0.07778	0.07760	0.29844	0.00000
Alterna	0.00000	0.00000	0.00000	0.00000	0.00000	0.00000	0.00000	0.27951	0.28985	0.20732	0.31108	0.23430	0.00000
Alterna	0.00000	0.00000	0.00000	0.00000	0.00000	0.00000	0.00000	0.22691	0.22514	0.28683	0.22534	0.15257	0.00000
Alterna	0.00000	0.00000	0.00000	0.00000	0.00000	0.00000	0.00000	0.17664	0.19923	0.17182	0.14514	0.14601	0.00000
Alterna	0.00000	0.00000	0.00000	0.00000	0.00000	0.00000	0.00000	0.15809	0.14014	0.16625	0.15036	0.09920	0.00000
Alterna	0.00000	0.00000	0.00000	0.00000	0.00000	0.00000	0.00000	0.06399	0.00000	0.09000	0.09048	0.06949	0.00000
Alterna	0.00000	0.00000	0.00000	0.00000	0.00000	0.00000	0.00000	0.00000	0.07572	0.00000	0.00000	0.00000	0.00000
Beliefs	0.17892	0.11778	0.16780	0.41972	0.11247	0.41053	0.42966	0.00000	0.00000	0.00000	0.00000	0.00000	0.00000
Confide	0.25966	0.17094	0.41972	0.28920	0.48856	0.32455	0.30745	0.00000	0.00000	0.00000	0.00000	0.00000	0.00000
Course	0.45760	0.30124	0.28920	0.16780	0.21929	0.15772	0.19745	0.00000	0.00000	0.00000	0.00000	0.00000	0.00000
Course	0.10381	0.41003	0.12328	0.12328	0.17968	0.10720	0.06544	0.00000	0.00000	1.00000	0.00000	0.00000	1.00000
Familia	0.00000	0.00000	0.00000	0.00000	0.00000	0.00000	0.00000	0.00000	1.00000	0.00000	0.00000	0.00000	0.00000
priorit	0.00000	0.00000	0.00000	0.00000	0.00000	0.00000	0.00000	0.00000	0.00000	0.00000	0.00000	0.00000	0.00000

Table 1 was converted into a weighted supermatrix after the normalization process. Table 2 shows the weighted supermatrix.

**TABLE 2:** Weighted supermatrix.

	Alterna	Alterna	Alterna	Alterna	Alterna	Alterna	Alterna	Beliefs	Confide	Course	Course	Familia	priorit
Alterna	0.00000	0.00000	0.00000	0.00000	0.00000	0.00000	0.00000	0.09486	0.03497	0.03889	0.07760	0.29844	0.00000
Alterna	0.00000	0.00000	0.00000	0.00000	0.00000	0.00000	0.00000	0.27951	0.14492	0.10366	0.31108	0.23430	0.00000
Alterna	0.00000	0.00000	0.00000	0.00000	0.00000	0.00000	0.00000	0.22691	0.11257	0.14341	0.22534	0.15257	0.00000
Alterna	0.00000	0.00000	0.00000	0.00000	0.00000	0.00000	0.00000	0.17664	0.09961	0.08591	0.14514	0.14601	0.00000
Alterna	0.00000	0.00000	0.00000	0.00000	0.00000	0.00000	0.00000	0.15809	0.07007	0.08313	0.15036	0.09920	0.00000
Alterna	0.00000	0.00000	0.00000	0.00000	0.00000	0.00000	0.00000	0.06399	0.00000	0.04500	0.09048	0.06949	0.00000
Alterna	0.00000	0.00000	0.00000	0.00000	0.00000	0.00000	0.00000	0.00000	0.03786	0.00000	0.00000	0.00000	0.00000
Beliefs	0.17892	0.11778	0.16780	0.41972	0.11247	0.41053	0.42966	0.00000	0.00000	0.00000	0.00000	0.00000	0.00000
Confide	0.25966	0.17094	0.41972	0.28920	0.48856	0.32455	0.30745	0.00000	0.00000	0.00000	0.00000	0.00000	0.00000
Course	0.45760	0.30124	0.28920	0.16780	0.21929	0.15772	0.19745	0.00000	0.00000	0.00000	0.00000	0.00000	0.00000
Course	0.10381	0.41003	0.12328	0.12328	0.17968	0.10720	0.06544	0.00000	0.00000	0.50000	0.00000	0.00000	1.00000
Familia	0.00000	0.00000	0.00000	0.00000	0.00000	0.00000	0.00000	0.00000	0.50000	0.00000	0.00000	0.00000	0.00000
priorit	0.00000	0.00000	0.00000	0.00000	0.00000	0.00000	0.00000	0.00000	0.00000	0.00000	0.00000	0.00000	0.00000

The weighted supermatrix was converted into the limit matrix for the process of prioritization of self-assurance of academic writing based on applied linguistics. Table 3 represents the limit matrix of the calculated comparison process.

**TABLE 3:** Limit matrix.

	Alterna	Alterna	Alterna	Alterna	Alterna	Alterna	Alterna	Beliefs	Confide	Course	Course	Familia	priorit
Alterna	0.05001	0.05001	0.05001	0.05001	0.05001	0.05001	0.05001	0.05001	0.05001	0.05001	0.05001	0.05001	0.05001
Alterna	0.11995	0.11995	0.11995	0.11995	0.11995	0.11995	0.11995	0.11995	0.11995	0.11995	0.11995	0.11995	0.11995
Alterna	0.09708	0.09708	0.09708	0.09708	0.09708	0.09708	0.09708	0.09708	0.09708	0.09708	0.09708	0.09708	0.09708
Alterna	0.07150	0.07150	0.07150	0.07150	0.07150	0.07150	0.07150	0.07150	0.07150	0.07150	0.07150	0.07150	0.07150
Alterna	0.06302	0.06302	0.06302	0.06302	0.06302	0.06302	0.06302	0.06302	0.06302	0.06302	0.06302	0.06302	0.06302
Alterna	0.02940	0.02940	0.02940	0.02940	0.02940	0.02940	0.02940	0.02940	0.02940	0.02940	0.02940	0.02940	0.02940
Alterna	0.00518	0.00518	0.00518	0.00518	0.00518	0.00518	0.00518	0.00518	0.00518	0.00518	0.00518	0.00518	0.00518
Beliefs	0.09076	0.09076	0.09076	0.09076	0.09076	0.09076	0.09076	0.09076	0.09076	0.09076	0.09076	0.09076	0.09076
Confide	0.13684	0.13684	0.13684	0.13684	0.13684	0.13684	0.13684	0.13684	0.13684	0.13684	0.13684	0.13684	0.13684
Course	0.11857	0.11857	0.11857	0.11857	0.11857	0.11857	0.11857	0.11857	0.11857	0.11857	0.11857	0.11857	0.11857
Course	0.14926	0.14926	0.14926	0.14926	0.14926	0.14926	0.14926	0.14926	0.14926	0.14926	0.14926	0.14926	0.14926
Familia	0.06842	0.06842	0.06842	0.06842	0.06842	0.06842	0.06842	0.06842	0.06842	0.06842	0.06842	0.06842	0.06842
priorit	0.00000	0.00000	0.00000	0.00000	0.00000	0.00000	0.00000	0.00000	0.00000	0.00000	0.00000	0.00000	0.00000

## Results and Discussion

**FIGURE 3:**
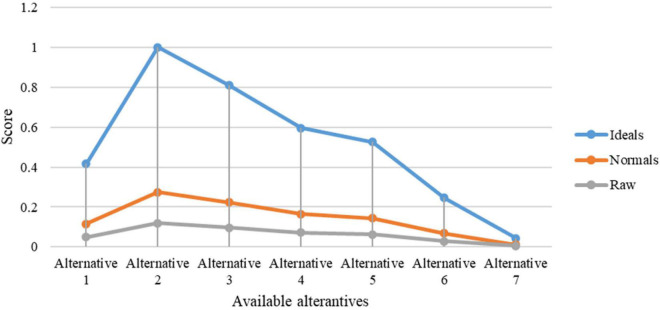
Representation of alternatives ranking.

The Super Decisions software was used for the purpose of experimental work. Initially, the hierarchy of goals, criteria, and alternatives was drawn for the process of the proposed research work. Then, the process of pairwise comparisons was done to derive the detailed calculations of the research. This section briefly shows the results and discussions of the current research work done. Higher education aims to assist students in realizing their full potential and achieving positive outcomes. Academic writing, for example, is employed to determine marks and contribute to degree course classification. Students’ expectations, baseline knowledge, and study and learning habits are all different. Students had different ideas about what constitutes academic writing for evaluation. Modern motives and beliefs emphasize the importance of students’ confidence in their studies. High self-esteem might motivate students to work harder to achieve tough goals. It may be more difficult for pupils to succeed in higher education if they are skeptical about their academic writing abilities. The current study has considered the DSS for prioritizing self-assurance of academic writing based on applied linguistics. More informed decision-making, timely problem-solving, and greater efficiency in dealing with issues, operations, planning, and even management are all possible with DSSs. Once all the matrices, including unweighted super matrix, super weighted matrix, and limit matrix are obtained, the prioritization process is shown. Figure 3 shows the ranking of alternatives from the perspectives of ideal, normal, and raw.

**FIGURE 4:**
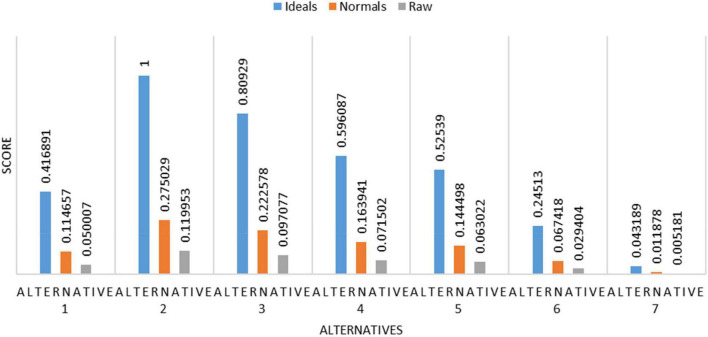
Overall synthesis priorities for the alternatives.

The overall synthesis priorities for the alternatives are shown in Figure 4. This figure shows that each alternative was checked for the case of “ideal,” “normal,” and “raw.” Thus, the process was done for all seven alternatives.

**FIGURE 5:**
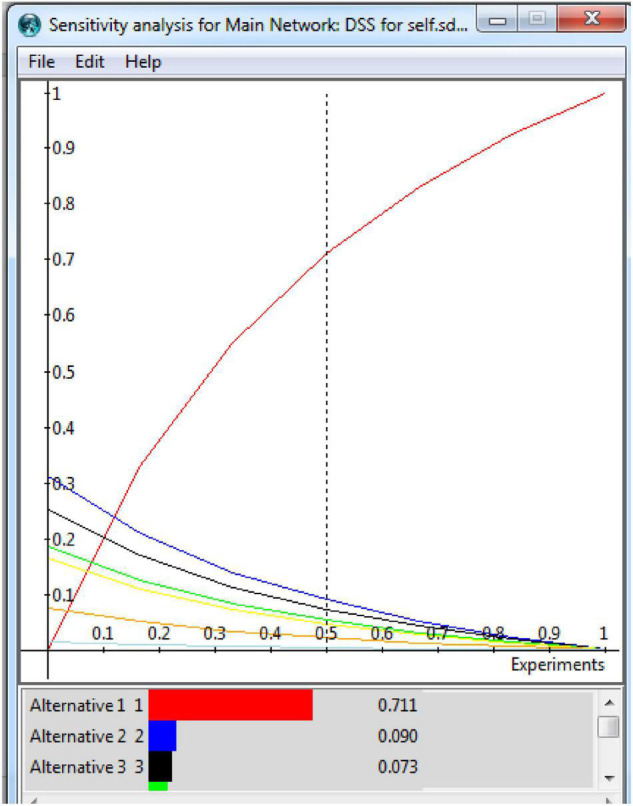
Analytic hierarchy process (AHP) sensitivity. Permission to reproduce images of Super Decisions has been given by Creative Decisions Foundation.

The analytic hierarchy process (AHP) sensitivity is shown in Figure 5. The sensitivity analysis is a basic idea in the implementation and efficient use of quantitative decision models. The purpose of sensitivity analysis is to evaluate the stability of an optimal solution under changes in the parameter. The following formula (Eq. 1) is used for the AHP preference (p_*i*_) of alternative (A_*i*_).


(1) pi=∑j=aijWj1N

where W_i_ is the weight of criteria (C_*yi*_) and a_ij_ is the preferred measure of alternative A.

With the help of the DSS, the data are filtered for easier management, which reduces the time between data gathering and decision-making for academic writing. Because the analytic process is systematic and each stage can be easily documented, DSSs improve early decision-making and enhance performance. After these experimental calculations, the analytic network process sensitivity was checked and is given. These were checked for all the available alternatives.

**FIGURE 6:**
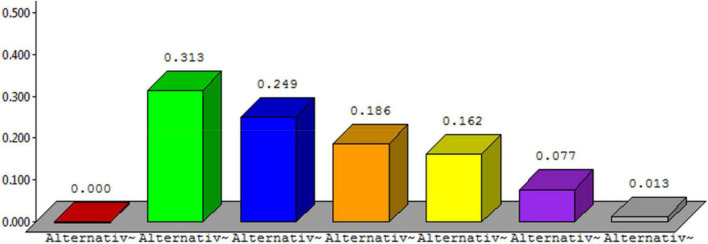
Alternative 1.

**FIGURE 7:**
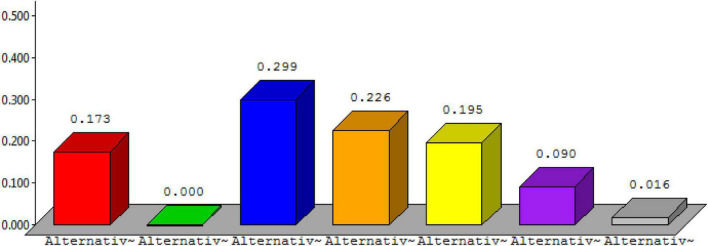
Alternative 2.

**FIGURE 8:**
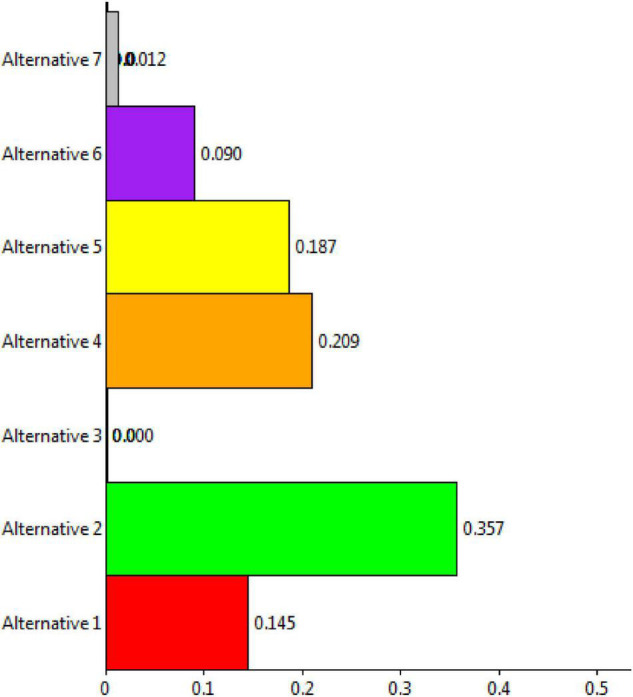
Alternative 3.

**FIGURE 9:**
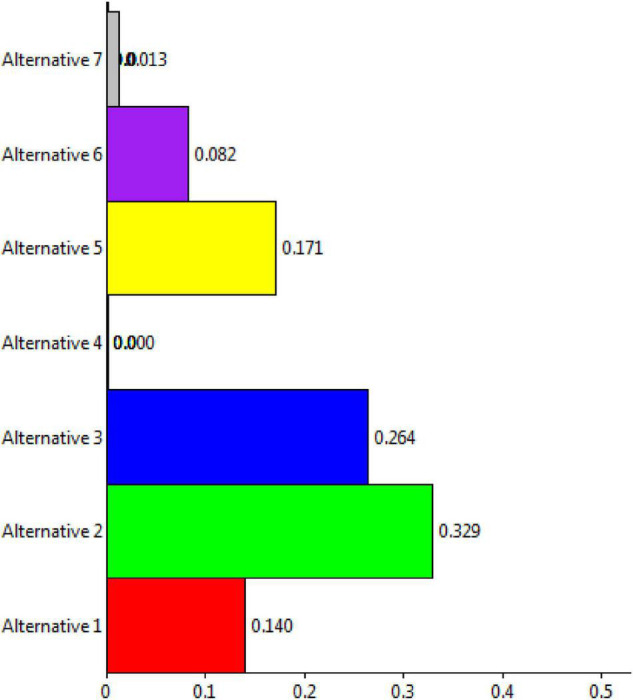
Alternative 4.

**FIGURE 10:**
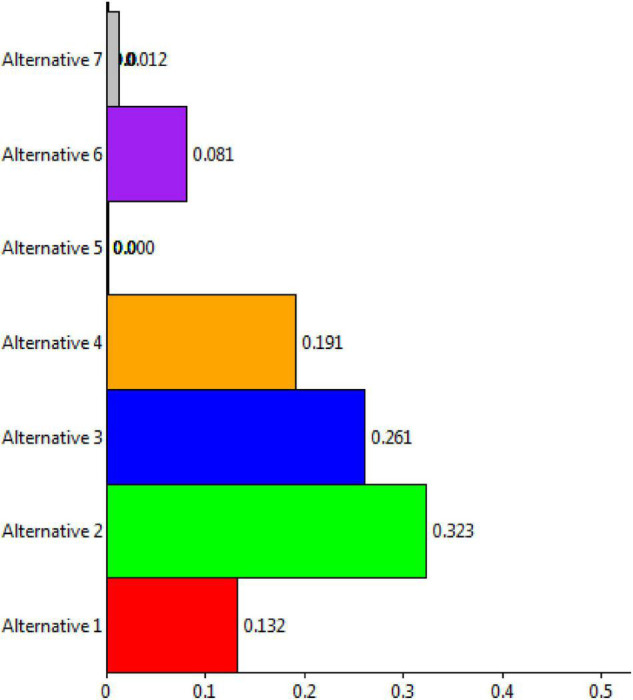
Alternative 5.

**FIGURE 11:**
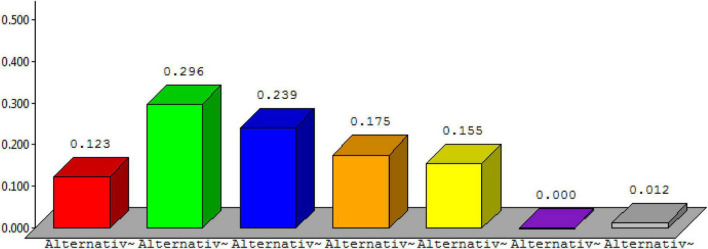
Alternative 6.

**FIGURE 12:**
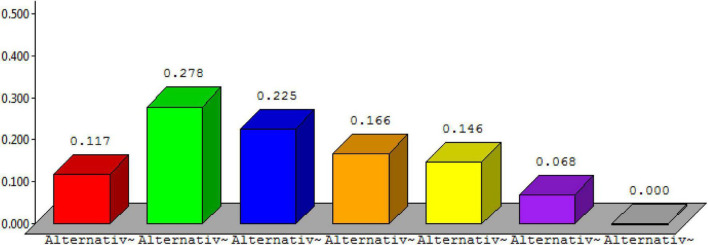
Alternative 7.

Below, Figure 6 describes the detail of alternative 1, Figure 7 describes the detail of alternative 2, Figure 8 describes the detail of alternative 3, Figure 9 describes the detail of alternative 4, Figure 10 describes the detail of alternative 5, Figure 11 describes the detail of alternative 6, and Figure 12 describes the detail of alternative 7.

**FIGURE 13:**
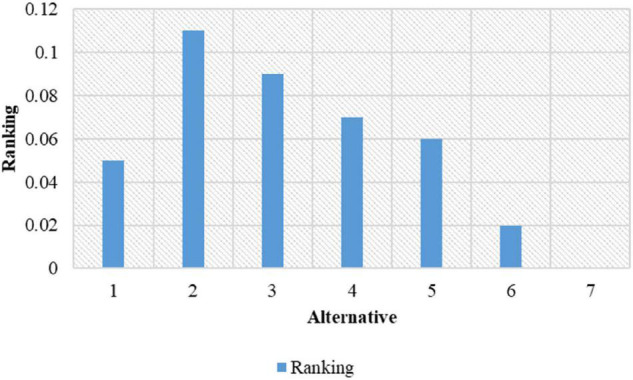
Overall ranking for the prioritization process.

The overall ranking for prioritization is shown in Figure 13. From the figure, it is shown that alternative 2 is having the topmost rank followed by alternative 3 and so on.

## Conclusion

English as a second language and EFL have been well-thought-out as fundamental thoughtfulness in evaluating and developing educational evaluation. However, research in these fields has shown that numerous factors affected these ESL and EFL reliability assessments.

Higher education and research institutions aim to sustain students in optimizing their prospects for achieving satisfactory outcomes. Since the last decade, various studies have been considered to identify score reliability and variability of assessing ESL and EFL. Modern-day motivations and theories emphasized the significance of students’ confidence within their studies. The role of high confidence can support students to apply more effort for setting challenging goals. The lower level of confidence about academic writing might be less supportive in higher education for students. A DSS has many applications in diverse areas and can play a significant role in ranking and prioritizing. Limitation of the proposed research is that only few features were considered. In the future, the existing feature of the study can be increased in order to get more beneficial and effective results. The proposed study contribution is to formulate a DSS in early decision-making based on the defined multicriteria for prioritizing self-assurance of academic writing in applied linguistics. The criteria and available alternatives for the self-assurance of academic writing were considered from the literature. The process of the proposed research was conducted in the Super Decision tool. Results of the study have shown the effectiveness of the proposed research.

## Data Availability Statement

The original contributions presented in the study are included in the article/supplementary material, further inquiries can be directed to the corresponding author.

## Author Contributions

Both authors performed the data mining and computing, contributed to the article and approved the submitted version.

## Conflict of Interest

The authors declare that the research was conducted in the absence of any commercial or financial relationships that could be construed as a potential conflict of interest.

## Publisher’s Note

All claims expressed in this article are solely those of the authors and do not necessarily represent those of their affiliated organizations, or those of the publisher, the editors and the reviewers. Any product that may be evaluated in this article, or claim that may be made by its manufacturer, is not guaranteed or endorsed by the publisher.

## References

[B1] Bahrami S. Touiserkani M. Momeni M. R. (2015). “An Examination of the Culture of Impartiality in Wikipedia, A Case Study of the Islamic World Representation in the English and Persian Versions of the Wikipedia,” in *2015 International Conference on Culture and Computing (Culture Computing)*, (Kyoto: IEEE), 113–118. 10.1109/Culture.and.Computing.2015.17

[B2] Barkaoui K. (2007). Rating scale impact on EFL essay marking: a mixed-method study. *Assess. Writ.* 12 86–107.

[B3] Bartlett T. (2020). No gods and precious few heroes: SFL and evolutionary linguistics. *Lingua* 261:102953. 10.1016/j.lingua.2020.102953

[B4] Brock A. (2017). Modelling the complexity of humour – insights from linguistics. *Lingua* 197 5–15. 10.1016/j.lingua.2017.04.008

[B5] Chen L. Hu G. (2020). Surprise markers in applied linguistics research articles: a diachronic perspective. *Lingua* 248:102992. 10.1016/j.lingua.2020.102992

[B6] Cheng F. W. Chiu M. C. (2018). Scaffolding chinese as a second language writing through a systemic functional linguistics approach. *System* 72 99–113. 10.1016/j.system.2017.11.003

[B7] Duan H. Y. (2015). “Research and implementation of intelligent linguistics and language teaching module based on network environment,” in *Proceedings of the 2014 International Conference on Computational Intelligence and Communication Networks, 14-16 Nov. 2014*, Bhopal, 492–495. 10.1109/CICN.2014.114

[B8] Farsani M. A. Jamali H. R. Beikmohammadi M. Ghorbani B. D. Soleimani L. (2021). Methodological orientations, academic citations, and scientific collaboration in applied linguistics: what do research synthesis and bibliometrics indicate?. *System* 100:102547. 10.1016/j.system.2021.102547

[B9] He X. L. Zhao H. Q. Zhong L. C. Nazir S. Khan A. S. (2021). Soft computing and decision support system for software process improvement: a systematic literature review. *Sci. Program.* 2021 1–14.

[B10] Janssen S. Schlaud D. (1999). “Culture and communication: a case study,” in *IPCC 99. Communication Jazz: Improvising the New International Communication Culture. Proceedings 1999 IEEE International Professional Communication Conference (Cat. No.99CH37023)*, (New Orleans: IEEE), 181–185. 10.1109/IPCC.1999.799119

[B11] Li Q. (2017). “An analysis of features of English for Science and Technology from the view of cognitive linguistics,” in *2017 international conference on advanced mechatronic systems (ICAMechS)*, (Xiamen: IEEE), 135–138. 10.1109/ICAMechS.2017.8316564

[B12] Lin L. Lee H. M. (2009). “An evaluation of survey by fuzzy linguistics based on the signed distance method,” in *2009 IEEE International Conference on Fuzzy Systems*, (Korea: IEEE), 1457–1461. 10.1109/FUZZY.2009.5277199

[B13] Liu Z. (2009). “The influence of organizational culture on language classroom,” in *2009 First International Workshop on Education Technology and Computer Science*, (Washington: IEEE), 52–57. 10.1109/ETCS.2009.535

[B14] Longhi J. (2021). Using digital humanities and linguistics to help with terrorism investigations. *Forensic Sci. Int.* 318:110564. 10.1016/j.forsciint.2020.110564 33218794

[B15] Marti L. Yilmaz S. Bayyurt Y. (2019). Reporting research in applied linguistics: the role of nativeness and expertise. *J. Engl. Acad. Purp.* 40 98–114. 10.1016/j.jeap.2019.05.005

[B16] Nazir S. Shahzad S. Mahfooz S. Nazir M. (2016). Fuzzy logic based decision support system for component security evaluation. *Int. Arab J. Inf. Technol.* 15 224–231.

[B17] Ren J. (2021). Variability and functions of lexical bundles in research articles of applied linguistics and pharmaceutical sciences. *J. Engl. Acad. Purp.* 50:100968. 10.1016/j.jeap.2021.100968

[B18] Riemer N. (2019). Cognitive linguistics and the public mind: idealist doctrines, materialist histories. *Lang. Commun.* 64 38–52. 10.1016/j.langcom.2018.09.002

[B19] Schwarz V. S. Hamman-Ortiz L. (2020). Systemic functional linguistics, teacher education, and writing outcomes for u.s. elementary english learners: a review of the literature. *J. Second Lang. Writ.* 49:100727. 10.1016/j.jslw.2020.100727

[B20] Sharifian F. (2017). Cultural linguistics and linguistic relativity. *Lang. Sci.* 59 83–92. 10.1016/j.langsci.2016.06.002

[B21] Shi J. (2021). “Phenomenon of Machine Mother Tongue Culture Transfer in English under the Internet with Pattern Recognition Models,” in *2021 International Conference on Artificial Intelligence and Smart Systems (ICAIS)*, (Coimbatore: IEEE), 1232–1235. 10.1109/ICAIS50930.2021.9395982

[B22] Shin S. Hashimoto H. Yoshida I. (2017). “Effects of Different Behaviors between Cross Cultures on Learners When Studying,” in *2017 International Conference on Culture and Computing (Culture and Computing)*, (Kyoto: IEEE), 82–88.

[B23] Su H. Zhang Y. Lu X. (2021). Applying local grammars to the diachronic investigation of discourse acts in academic writing: the case of exemplification in linguistics research articles. *Engl. Specif. Purp.* 63 120–133. 10.1016/j.esp.2021.05.002

[B24] Sun L. Nazir S. Hussain A. (2021). Multi-criteria decision making to continuous software improvement based on quality management, assurance and metrics. *Sci. Program.* 2021:9953618. 10.1155/2021/9953618

[B25] Tsepilova A. V. Mikhaleva L. V. (2015). Working with formulaic language as a way to evaluate and improve efl non-linguistics students’ pragmatic skills in a culture-specific contextual situation. *Procedia Soc. Behav. Sci.* 200 550–556. 10.1016/j.sbspro.2015.08.022

[B26] Yamaguchi A. Koyama W. (2009). Toward a critical dialogue across languages and cultures: on native and western linguistics in modern japan. *J. Pragmat.* 41 147–156. 10.1016/j.pragma.2008.09.009

[B27] Yang M. Nazir S. Xu Q. Ali S. (2020). Deep learning algorithms and multicriteria decision-making used in big data: a systematic literature review. *Complexity* 2020:2836064.

[B28] Yanping Y. Zhenhua C. (2020). “Integrating Chinese Culture in College English Course,” in *2020 International Conference on Modern Education and Information Management (ICMEIM)*, (Dalian: IEEE), 820–823. 10.1109/ICMEIM51375.2020.00182

[B29] Zhang J. Nazir S. Huang A. Alharbi A. (2020). Multicriteria decision and machine learning algorithms for component security evaluation: library-based overview. *Secur. Commun. Netw.* 2020 1–14.

[B30] Zotzmann K. Sheldrake R. (2021). Postgraduate students’ beliefs about and confidence for academic writing in the field of applied linguistics. *J. Second Lang. Writ.* 52:100810. 10.1016/j.jslw.2021.100810

